# Blood flow restriction combined with resistance training on muscle strength and thickness improvement in young adults: a systematic review, meta-analysis, and meta-regression

**DOI:** 10.3389/fphys.2024.1379605

**Published:** 2024-08-12

**Authors:** Feier Ma, Jianghua He, Yan Wang

**Affiliations:** School of Sports Medicine and Rehabilitation, Beijing Sport University, Beijing, China

**Keywords:** resistance training (RT), muscle strength, muscle thickness, blood flow restriction (BFR), young adults

## Abstract

**Background:**

High-intensity resistance training is known to be the most effective method for enhancing muscle strength and thickness, but it carries potential injury risks. Blood flow restriction (BFR) combined with resistance training has been proposed as a safer alternative method for improving muscle strength and thickness.

**Methods:**

A meta-analysis was conducted, including 20 studies from five databases that met the inclusion criteria, to assess the efficacy of BFR combined with resistance training compared to traditional resistance training (NOBFR). The analysis focused on changes in muscle strength and thickness. Subgroup analysis and meta-regression were performed to explore the effects of tourniquet width and pressure.

**Results:**

The findings showed that BFR combined with resistance training is comparable to traditional resistance training in enhancing muscle strength [0.11, 95%CI: (−0.08 to 0.29), *I* ^*2*^ = 0%] and muscle thickness [−0.07, 95% CI: (−0.25 to 0.12), *I* ^*2*^ = 0%]. Subgroup analysis indicated no significant differences in muscle strength (P = 0.66) and thickness (P = 0.87) between low-intensity BFR training and other intensity levels. Meta-regression suggested that tourniquet width and pressure might affect intervention outcomes, although the effects were not statistically significant (P > 0.05).

**Conclusion:**

BFR combined with resistance training offers a viable alternative to high-intensity resistance training with reduced injury risks. We recommend interventions of 2-3 sessions per week at 20%–40% of 1 RM, using a wider cuff and applying an arterial occlusion pressure of 50%–80% to potentially enhance muscle strength and thickness. It is also recommended to release tourniquet pressure during rest intervals to alleviate discomfort. This protocol effectively improves muscle strength with minimal cardiac workload and reduced risk of adverse events.

**Systematic Review Registration:**

[https://www.crd.york.ac.uk/prospero/display_record.php?ID=CRD42023495465], identifier [CRD42023495465].

## 1 Introduction

Muscle, as a major component of the locomotor system, muscle mass determines the athletic capability and sports performance (van der Zwaard et al., 2021; Khare et al., 2023). Muscle mass is critical for both athletes and chronically ill people. For athletes, muscle mass determines performance to influence competitive results. For chronically ill or elderly populations, muscle mass correlates with longevity (Cruz-Jentoft and Sayer, 2019; Fabero-Garrido et al., 2022a; Alizadeh Pahlavani, 2022). Traditional resistance training has been validated as an effective non-pharmacological intervention for enhancing muscle mass and strength (Fragala et al., 2019). High-intensity (HI) resistance training has demonstrated superior effect on muscle strength and thickness improvement compared to moderate-to low-intensity (LI) resistance training (Csapo and Alegre, 2016). However, it is imperative to note that HI resistance training may induce pain and injuries to populations with chronic disease, such as hypertension or osteoarthritis (Wang et al., 2022a; Wang et al., 2022b). Consequently, it is crucial to explore alternative approaches that yield benefits akin to HI resistance training while mitigating associated risks.

Recent studies have revealed that LI resistance training, when combined with blood flow restriction (BFR), triggers heightened metabolic stress, thereby modulating signal transduction in musculoskeletal cells and achieving muscle strength and thickness improvements comparable to those achieved through HI resistance training (Krzysztofik et al., 2019; Koc et al., 2022; Krzysztofik et al., 2022). BFR is an intervention method that involves the application of restrictive equipment to reduce blood flow in the proximal segment of the limb (Lorenz et al., 2021). This intervention indirectly influences cellular metabolism by accumulating metabolites and simulates a localized hypoxic environment during the reduction of blood flow in the proximal limb segment (Krzysztofik et al., 2019; Lorenz et al., 2021). The combination of BFR with resistance exercise has been implemented across diverse populations in both clinical and non-clinical setting (Krzysztofik et al., 2019; Krzysztofik et al., 2022; Colapietro et al., 2023). Consequently, BFR coupled with LI resistance training emerges as a safe alternative treatment, presenting a viable option to traditional resistance training protocols for enhancing muscle and physical functions among patients in the clinical setting or sedentary populations.

Despite the growing utilization of BFR in combination with resistance training in various studies and clinical settings, there remains a conspicuous absence of standardized criteria regarding training protocols and BFR equipment among participants. Key parameters such as occlusion pressure, cuff width, and the choice of resistance level are pivotal in determining the effectiveness of BFR interventions. Variations in treatment protocols, including occlusion pressure [often recommended to be between 50%–80% of arterial occlusion pressure (Li et al., 2023)], cut width, the number of sets and repetitions performed, total training sessions, and duration, may contribute to differences in the treatment’s efficacy within clinical applications (Brumitt et al., 2021a). Moreover, a dearth of evidence exists to comprehensively evaluate the impact of BFR combined with resistance training at various intensity levels on improving muscle strength and thickness. This study employs meta-regression analysis to evaluate the impact of these parameters on muscle strength and thickness improvement, aiming to provide a clearer understanding of how different training and equipment characteristics affect outcomes. The influence of training and equipment characteristics on muscle function improvement remains a crucial area requiring further exploration and in-depth discussion.

This study aims to address these gaps by examining the influence of treatment characteristics on muscle strength and thickness improvement through a systematic review and meta-analysis. A subsequent meta-regression analysis intends to rigorously investigate the association between specific protocol details and muscular improvement. The insights gleaned from this study not only contribute to the current understanding of resistance training with BFR but also furnish valuable information for designing effective and targeted treatment programs.

## 2 Method

This review was prospectively registered with PROSPERO (ref. CRD42023495465) and was reported in accordance with the Preferred Reporting Items for Systematic Reviews and Meta-Analyses (PRISMA) guidelines (Moher et al., 2009) and the Cochrane Handbook (JPT et al., 2024).

The screening of studies, quality assessment, and data extraction were independently conducted by two researchers, FM and JH. Any discrepancies in assessments were resolved through discussion. In instances where a consensus could not be reached, a third researcher, YW, was consulted to facilitate an agreement.

### 2.1 Data sources and study selection

The search was initiated on 8 October 2023, using multiple databases including PubMed, Web of Science, EBSCO, Embase, and the Cochrane Library. The search involved keywords such as “*Blood Flow Restriction,*” “*hypoxia,*” and “*resistance training,*” and was limited to studies published between 1985 and 2023. The detailed search strategies for each database are summarized in Table 1.

**TABLE 1:** Database search strategies.

Database	Search Strategy
PubMed	(“Resistance Training” [MeSH Terms] OR “strength training” [Title/Abstract] OR “resistance exercise” [Title/Abstract] OR “weight training” [Title/Abstract]) AND (“Hypoxia” [MeSH Terms] OR “altitude” [Title/Abstract] OR “hypoxic training” [Title/Abstract] OR “hypoxic exposure” [Title/Abstract]) AND (“Blood Flow Restriction Therapy” [MeSH Terms] OR “karats” [Title/Abstract] OR “occlusion training” [Title/Abstract] OR “blood flow restriction” [Title/Abstract] OR “br training” [Title/Abstract] OR “br exercise” [Title/Abstract])
Web of Science	TS=(“Resistance Training” OR “strength training” OR “resistance exercise” OR “weight training”) AND TS=(“Hypoxia” OR “altitude” OR “hypoxic training” OR “hypoxic exposure”) AND TS=(“Blood Flow Restriction Therapy” OR “karats” OR “occlusion training” OR “blood flow restriction” OR “br training” OR “br exercise”)
EBSCO	(“Resistance Training” OR “strength training” OR “resistance exercise” OR “weight training”) AND (“Hypoxia” OR “altitude” OR “hypoxic training” OR “hypoxic exposure”) AND (“Blood Flow Restriction Therapy” OR “karats” OR “occlusion training” OR “blood flow restriction” OR “br training” OR “br exercise”)
Embase	(‘Resistance Training’/exp OR ‘strength training’ OR ‘resistance exercise’ OR ‘weight training’) AND (‘Hypoxia’/exp OR ‘altitude’ OR ‘hypoxic training’ OR ‘hypoxic exposure’) AND (‘Blood Flow Restriction Therapy’/exp OR ‘karats’ OR ‘occlusion training’ OR ‘blood flow restriction’ OR ‘br training’ OR ‘br exercise’)
Cochrane Library	(“Resistance Training” OR “strength training” OR “resistance exercise” OR “weight training”) AND (“Hypoxia” OR “altitude” OR “hypoxic training” OR “hypoxic exposure”) AND (“Blood Flow Restriction Therapy” OR “karats” OR “occlusion training” OR “blood flow restriction” OR “br training” OR “br exercise”)

An example of the study search strategy and the results obtained using the PubMed database is provided in Supplementary Appendix Table S1 *.*

Prior to commencing the screening process, studies were included if they met the following criteria: 1) participants were healthy adults aged 18+ years; 2) the intervention involved resistance training combined with BFR; 3) the intervention of control group received usual care without BFR (NOBFR); 4) the study outcomes centered on muscle thickness (cross-sectional area (CSA) or girth) and muscle strength (1 repetition maximum (RM) or maximal voluntary torque (MVT)); 5) the studies were randomized controlled trials; 6) the studies were available in English.

Studies were exclued, if: 1) participants had chronic diseases or pain, such as hypertension; 2) the intervention was acute exercise and aerobic training with BFR; 3) the studies were observational or cohort trails, conference reports, and review articles; 4) the studies were published in a language other than English.

The searched studies were evaluated against these criteria in two phases: 1) assessment of each study’s abstract and title and 2) evaluation of the full text of potentially relevant studies. The search and screening process was documented using the PRISMA flow diagram (Page et al., 2021). The researchers used Endnote software for the screening process.

### 2.2 Quality assessment and data extraction

#### 2.2.1 Assessment of bias

The methodological quality of the included studies underwent rigorous assessment by the researchers using two tools: the Cochrane Risk of Bias 2 (RoB 2) tool (Higgins et al., 2011) and the PEDro (Physiotherapy Evidence Database) scale (Maher et al., 2003). Each tool’s guidelines were followed during the assessment process, evaluating five key domains: the randomization process, confounding factors, sample selection, missing data, and measurement of outcomes. Utilizing the Cochrane RoB 2 tool, the risk was classified as “low,” “some concerns,” or “high,” providing a detailed overview of the methodological quality of the included studies. Additionally, the 11-item PEDro scale (http://www.pedro.fhs.usyd.edu.au) was employed to gauge methodological quality (de Morton, 2009), with items scored as either present (“1”) or non-present (“0”), allowing for a comprehensive assessment of each study. The methodological quality was categorized as “low” (total score less than 4), “some concerns,” and “high” (total score greater than 8). The evaluation results are detailed in Figure 2; Table 1. This rigorous approach ensures the reliability and validity of the meta-analysis findings.

#### 2.2.2 Data extraction and synthesis

Data extracted from each included study were divided into two categories: participant characteristics and trial characteristics. Participant characteristics encompassed crucial information such as sample age, body weight, BMI, and sample size. It is noted that several studies included multiple intervention groups and self-control groups. To prevent duplication of samples, the sample size of self-control studies was averaged equally. For studies with different intervention groups, each intervention was reported separately, and the control group’s sample size was also equally distributed. Trail characteristics encompassed training intensity, tourniquet width, occlusion pressure, tourniquet application time, training volume, rest duration between sets, training duration, frequency, and the specific outcomes obtained in each study. Continuous numerical data were extracted using mean and standard deviation (SD). Further details regarding the extracted data are showed in Table 2. Investigation into the effect of trial characteristics of BFR intervention on muscle strength and thickness improvement necessitated the transformation of certain trail characteristics into binary variables for data analysis. Specifically, the training intensity with BFR was dichotomized into “Low-intensity with BFR” (50% 1 RM) or “Other intensity with BFR” [including middle-(50–69% 1RM) and high-intensity with BFR (70–84% 1RM)]. The inflation of the tourniquet during exercise interventions was categorized as either “Inflated for the entire exercise protocol” or “Inflated during exercise and deflated during rest periods.”

**TABLE 2:** Study quality assessment using the PEDro scale.

Study	1	2	3	4	5	6	7	8	9	10	11	Overall	Quality
Barcelos et al. (2015)	1	1	1	1	0	0	0	1	1	1	1	8/11	High
Biazon et al. (2019)	1	1	1	1	0	0	0	1	1	1	1	8/11	High
Bradley et al. (2022)	1	1	1	1	0	0	1	0	1	1	1	8/11	High
Brumitt et al. (2021c)	1	1	1	1	0	0	1	1	1	1	1	9/11	High
Centner et al. (1985)	1	1	1	1	0	0	1	1	1	1	1	9/11	High
Cook and Cleary (2019)	0	1	1	1	0	0	0	1	1	1	1	7/11	Some concern
Colapietro (2023)	1	1	1	1	0	0	0	1	1	1	1	8/11	High
Fahs et al. (2015)	1	1	1	1	0	0	0	0	1	1	1	7/11	Some concern
Fernandes et al. (2020)	1	1	1	1	0	0	0	1	1	1	1	8/11	High
Hackney et al. (2016)	1	0	1	1	0	0	0	1	1	1	1	7/11	Some concern
Kacin and Strazar (2011)	1	0	1	1	0	0	0	1	1	1	1	7/11	Some concern
Laurentino et al. (2008)	1	0	1	1	0	0	0	1	1	1	1	7/11	Some concern
Laurentino et al. (2022)	1	1	1	1	0	0	0	1	1	1	1	8/11	High
Lixandrão et al. (2015)	1	0	1	1	0	0	0	0	1	1	1	6/11	Some concern
Madarame et al. (2008)	0	1	1	1	0	0	0	1	1	1	1	7/11	Some concern
Ozaki et al. (2013)	1	1	1	1	0	0	0	1	1	1	1	8/11	High
Reece et al. (1985)	1	1	1	1	0	0	0	1	1	1	1	8/11	High
Teixeira et al. (2022)	1	1	1	1	0	0	0	0	1	1	1	7/11	Some concern
Vechin et al. (2015)	1	0	1	1	0	0	0	1	1	1	1	7/11	Some concern
Yasuda et al. (2014)	1	1	1	1	0	0	0	1	1	1	1	8/11	High

For the meta-analysis and meta-regression analysis, this study utilized the mean and SD of the post-intervention test results for each study’s intervention and control groups. The outcomes measured included including muscle CSA and girth, as well as muscle strength (including 1 RM and MVT). Notably, the meta-analysis was conducted separately for muscle thickness and muscle strength.

### 2.3 Data analysis

The initial meta-analysis of the extracted data took into consideration the methodological heterogeneity arising from diverse muscle measurement and testing methods. To address this, a standardized mean difference (SMD) (Bakbergenuly et al., 2020) was employed for data analysis. This approach utilizes statistical units to standardize various clinical units, effectively reducing discrepancies caused by differing testing methods and clinical units (Andrade, 2020). The SMD calculation is the following:

$$\text{SMD}=\left({\text{Mean}}_{\text{BFR}}\;-{\text{Mean}}_{\text{Non}-\text{BFR}}\right)÷{\text{SD}}_{\text{BFR}}$$

Following this, the present study evaluated the heterogeneity of the included studies’ results using Cochran’s Q statistic (Schulzke and Patole, 2021), a well-established tool for accurately gauging statistical heterogeneity, alongside Higgins and Thompson’s *I* ^*2*^ statistic to quantify the heterogeneity level (Bowden et al., 2011). Meanwhile, forest plots were used for visually representation of the analysis results. The *I* ^*2*^ statistic was used to quantify the degree of heterogeneity across studies, with values of 25%, 50%, and 75% denoting low, moderate, and high heterogeneity (Higgins et al., 2003), respectively. In instances where the *I* ^*2*^ value exceeded 75%, indicating significant inconsistency among these studies, a reassessment was conducted using a random-effects model. After that, publication bias and sensitivity analyses were performed to comprehend the reasons behind the observed heterogeneity. Specifically, to evaluate the potential for publication bias, a meta-bias assessment was conducted in the study. A funnel plot was constructed and the Egger’s test was used to detect statistically significant publication bias (P < 0.1) (JPT et al., 2024). Furthermore, a sensitivity analysis was executed to explore the impact of each study on bias. Studies demonstrating high inconsistency were considered for potential exclusion in the final analysis. Researchers assigned weights to each included study, where studies with high inconsistency were weighted as 0 while others were weighted as 1 for the formal analysis.

### 2.4 Meta-regression analysis

This study conducted a meta-regression analysis to delve into the relationship between trial characteristics and the improvement of muscle strength and thickness. Meta-regression enables the exploration of potential sources of heterogeneity by quantifying the impact of various factors on intervention effects. The Knapp-Hartung modification was incorporated to bolster the robustness of the analysis. The primary objective of this analysis was to discern significant associations between individual trial characteristics and the outcomes, thereby offering valuable insights into the factors influencing muscle response across the included studies.

### 2.5 Statistical analysis

This study employed a combination of software tools to execute various aspects of the data analysis process. Endnote (Clarivate, Philadelphia, United States) was utilized for reference management and the screening process. The methodological quality assessment of the included studies was conducted using Excel to implement RoB 2, and the *Robvis* package (McGuinness and Higgins, 2020) in R Studio (R version 4.2.3; RStudio, PBC, Boston, MA, United States) was adopted for visualizing the RoB assessment. The results were exhibited in two figures: a summary of five domains and a traffic light figure displaying each study’s risk. R studio served as the primary tool for conducting the meta-analysis, publication bias analysis, sensitivity analysis, and figure generation. Stata 18 (StataCorp, College Station, TX, United States) was utilized specifically for the meta-regression analysis to explore the relationships between moderators and outcomes. All results were calculated with 95% confidence intervals (CI). For the determination of statistical significance in BFR intervention effects, the threshold was set at *P* < 0.05. In addition, statistical significance for heterogeneity was defined as *P* < 0.1 or *I* ^*2*^ > 75%, and that for publication bias was defined as *P* < 0.1.

## 3 Results

### 3.1 Search results

**FIGURE 1:**
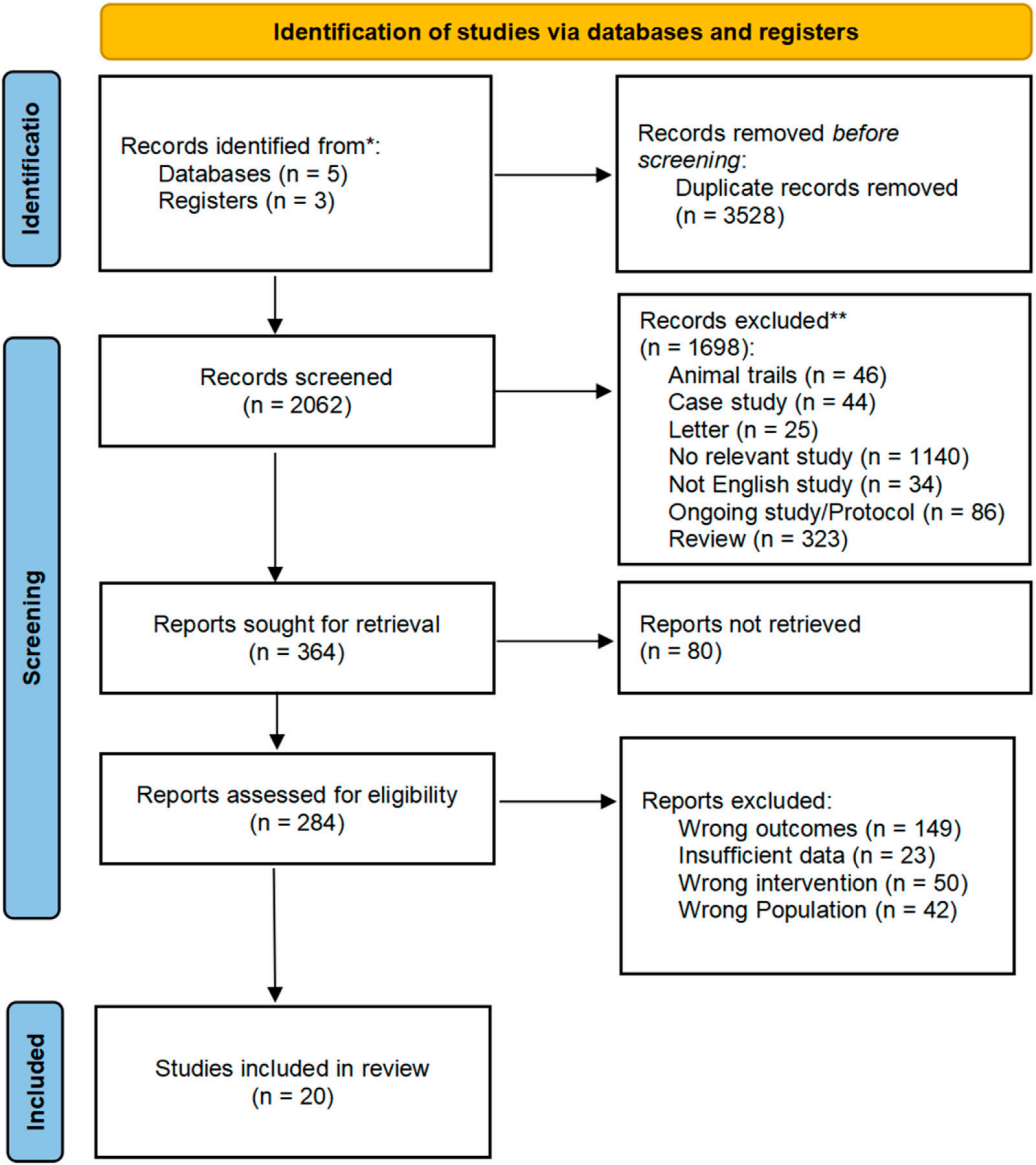
The PRISMA flow diagram of studies selection process.

The study identified 20 randomized controlled trails that met the specified inclusion criteria. Comprehensive searches using keywords across five databases yielded a total of 5,590 literature records. Following a careful screening process, 3,528 duplicate studies were removed, employing both automated and manual procedures facilitated by Endnote. Following this, three researchers conducted an initial screening of the included studies based on the predetermined criteria, assessing the title and abstract of each literature record. Among these, 80 records were excluded due to incomplete reporting of the study, and 284 records were screened in full text. During the full-text assessment, 23 articles were excluded due to insufficient content, as they only included change data between pre-training and post-training without detailed experimental results. Additionally, 50 studies were excluded due to inappropriate intervention for this review, such as BFR combined with aerobic training or walking trials, and 42 trials were excluded due to inappropriate participants, such as pre- and post-knee surgery patients. The detailed screening process is illustrated in Figure 1.

### 3.2 Risk assessment for study quality

**FIGURE 2:**
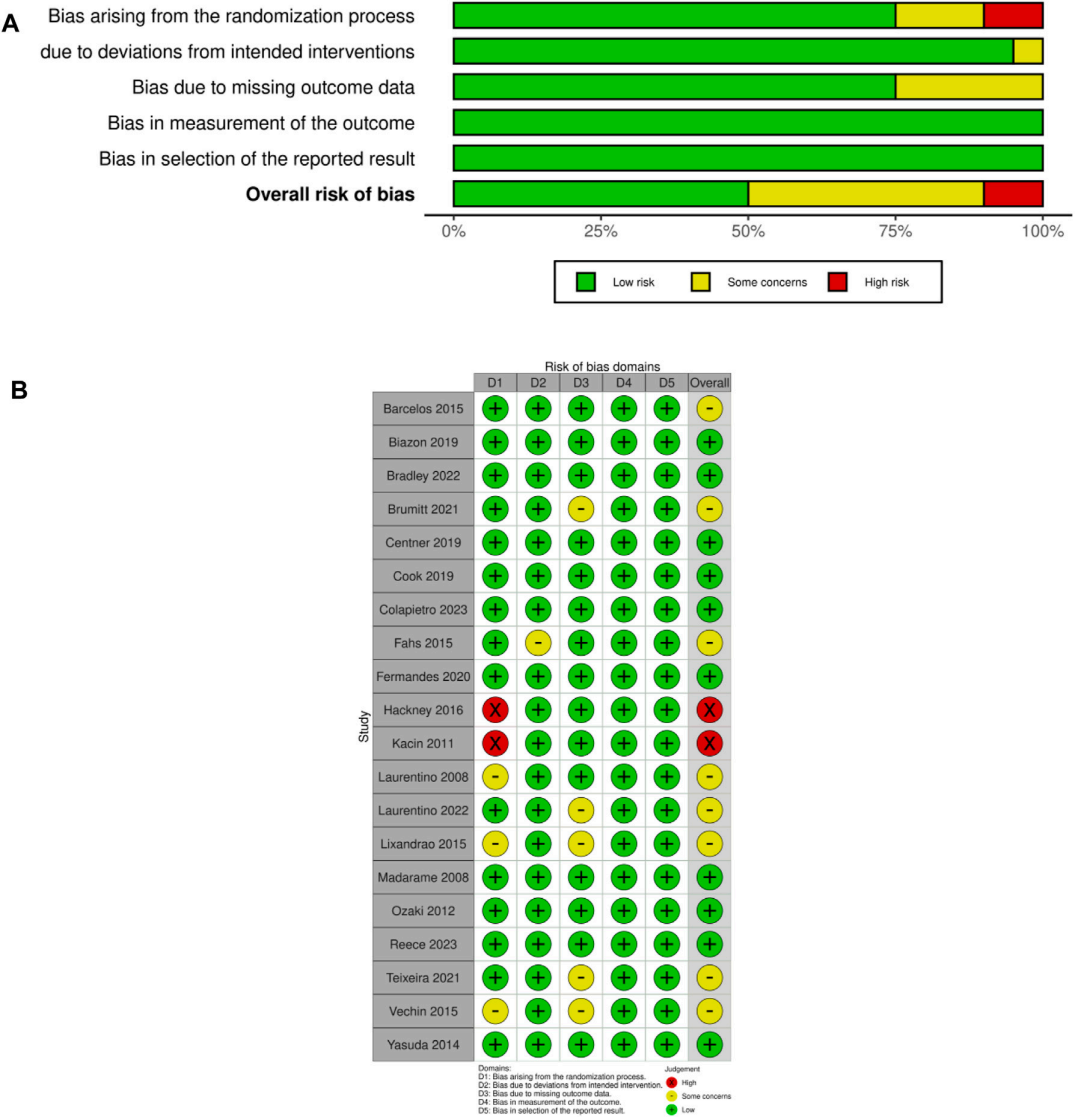
**(A)** RoB 2 summary plot; **(B)** RoB 2 traffic light figure. Note: The figures were produced using R packages (McGuinness and Higgins, 2020).

The methodological quality of the included studies has been meticulously assessed and is presented in Figure 2; Table 2. According to the RoB 2 assessment, ten studies (50%) were determined to exhibit a low risk of bias in their methodology, while only two studies (10%) were deemed to have a high risk. The remaining studies were found to have some concerns regarding their methodological quality. In addition, the PEDro scale scores of the included studies ranged from 6 to 9, with an average score of 7.6 ± 0.75. This range indicated a predominantly high methodological quality across the selected studies. Specifically, nine studies (45%) exhibited some concerns with their methodological quality, while five studies (25%) failed to employ random allocation groups. It is noteworthy that only one study (Lixandrão et al., 2015) scored 6 points and was assessed as having some concerns at RoB 2. This particular study might have contributed to the observed heterogeneity and potentially influenced the overall robustness of the outcomes.

### 3.3 Participant and trial characteristics

A total of 28 intervention groups from the 20 studies were reported in the analysis. The overall meta-analysis of the 20 studies included 515 participants, with study sizes varying from 4 to 19 subjects. Participant demographics revealed an age of 29.92 ± 2.22 (95% CI, 25.56–34.27), an body weight of 69.97 ± 1.59 (95% CI, 66.85–73.08, Q statistic = 52.75, *P* for heterogeneity = 0.07), and a BMI of 23.89 ± 0.66 (95% CI, 22.60–25.18, Q statistic = 65.98, *P* for heterogeneity <0.0001). Table 3 shows the participant characteristics and trials characteristics of the 20 included studies that met the selection criteria.

**TABLE 3:** Summary of participant characteristics and trial characteristics of included studies.

Study	Participant characteristics				Trial characteristics								
	Age (yr)	Body weight (kg)	BMI [kg·m]	Sample Size	Intervention	Tourniquet (width * length, cm)	The occlusion pressure	Tourniquet application time	Training protocol	Rest	Duration (weeks)	Frequency (per week)	Outcomes
Barcelos et al. (2015)	21 ± 4.76		23.3 ± 3.39	10	50% 1-RM	10 * 80	200 mmHg	Inflated for the entire exercise protocol	76	1 min between set	8	2	The cross-sectional area (CSA) of the quadriceps 1RM
	22 ± 2.9		22.4 ± 4.44	10	20% 1-RM	10 * 80	200 mmHg		25				
	21 ± 3.23		25.0 ± 6.05	10	20% 1-RM	10 * 80	200 mmHg		42				
Biazon et al. (2019)	22 ± 3	72.7 ± 10.7	22.81 ± 2.99	10	80% 1-RM	NA	NA	-	3 * 10	1 min between set	10	2	Unilateral quadriceps maximum dynamic strength; vastus lateralis (VL) muscle CSA; echo intensity; pennation angle (PA)
				10	80% 1-RM	17.5 * 92	60% of occlusion pressure (81.85 ± 4.45 mmHg)	Inflated during the exercise and deflated during the rest periods	3 * 10				
				10	20% 1-RM				3 * 20				
Bradley et al. (2022)	28.8 ± 6.3	67.0 ± 8.4		10	80% 1-RM(Rowing) 60% 1-RM(Deadlift)	-	-	-	Rowing: 3 * 1 min Deadlift: 20/10/10/10	0.5 min between set; 3 min between exercise	4	2	VL and biceps femoris (BF) CSA; 1-RM of deadlift; Thigh circumference; maximal aerobic capacity (VO2max)
	29.2 ± 8.0	80.4 ± 22.6		10	40% 1-RM(Rowing) 30% 1-RM(Deadlift)	11.43 * 86.36	-	Inflated for the entire deadlift protocol	Rowing: 3 * 1 min Deadlift:10/5/5/5				
Brumitt et al. (2021c)	25.8 ± 1.6			17	30% 1RM	-	-	-	30/15/15/15 (the single-leg knee extension exercise) 30/15/15/15 (the standing single-leg hamstring curl)	0.5 min between set	8	2	supraspinatus, shoulder ER, quadriceps, and hamstrings strength quadriceps CSA.
					30% 1RM	-	80% LOP	Inflated for the entire lower extremities protocol					
Centner et al. (1985)	26.1 ± 4.2	76.4 ± 15.4	23.5 ± 3.5	14	70%–85% 1RM	-	-	-	3 * 6–12	1 min between set; 3 min between exercise	14	3	CSA; Unilateral isometric maximum voluntary contraction (MVC); Achilles Tendon Properties; Lifestyle Parameters
	27.1 ± 4.7	85.0 ± 9.3	26.3 ± 3.5	11	20%–35% 2RM	12	50% AOP	Inflated during the exercise and deflated during the rest periods	30/15/15/15				
Cook and Cleary (2019)	76.3 ± 8.7	73.3 ± 10.9	26.5 ± 3.0	11	70% 1RM	-	-	-	Volitional failure (HL-Knee extension 23.5 ± 5.0)	1 min between set; 3 min between exercise	12	2	CSA; Strength (Unilateral, isometric maximum voluntary contraction (MVC))
	76.4 ± 6.6	75.4 ± 10.9	27.5 ± 3.3	10	30% 1RM	6 * 83	66% of predicted arterial occlusion pressure	Inflated during the exercise and deflated during the rest periods	Volitional failure (BFR-Knee flexion 18.1 ± 8.3)				
Colapietro (2023)	22.9 ± 3.78	70.1 ± 7.72	24.7 ± 1.82	10	50%–80% 1RM	-	-	-	3 * 8	0.5 min rest between sets; 1 min rest between exercises	4	3	Eccentric knee flexor peak moment; Rate of perceived exertion (RPE); CSA.
	20.7 ± 2.36	68.6 ± 7.23	24.3 ± 1.54	10	10%–30% 1RM	11.43 * 86.36	80% LOP	Inflated during the exercise and deflated during the rest periods	30/15/15/15	0.5 min rest between sets; 2 min rest between exercises			
Fahs et al. (2015)	55 ± 7	82.7 ± 16.5	26.7 ± 4.7	17	30% 1RM	-	-	-	Volitional failure (45 ± 15 repetitions)	NA	6	3	Muscle thickness (MTh); muscle strength (1 RM); Thigh circumference
					30% 1RM	5	80% of AOP but no higher than 240 mmHg	Inflated for the entire exercise protocol	Volitional failure (44 ± 13 repetitions)				
Fernandes et al. (2020)	20.2 ± 1.1	62.3 ± 6.9	23.3 ± 2.7	14	65%–85% 1RM	-	-	-	3 * 8–12	0.5 min rest between sets	4	3	Circumference; hand pressure strength
	20.1 ± 1.6	60.6 ± 8.6	23.1 ± 3.6	14	30%–55% 1RM	7 * 80	160 mmHg	Inflated for the entire exercise protocol	3 * 15–25				
Hackney et al. (2016)	33.8 ± 13.8	70.2 ± 17.0	70.2 ± 17.0	6	70%–80% 1RM	-	-	-	Volitional failure (3 * nr)	1.5 min rest between exercises	3.5 (25 days)	3	CSA; 1RM.
	30.1 ± 12.1	66.7 ± 6.7	66.7 ± 6.7	7	20%–30% 1RM	6 * 83	140 ± 10 mmHg	Inflated for the entire exercise protocol					
Kacin and Strazar (2011)	22.5 ± 0.6	76.7 ± 3.1		10	15% MVC force	-	-	-	Volitional failure (4 * nr)	2 min between set.	4	4	Muscle CSA; MVC force; EMG; Muscle oxygen; Arterial blood pressureHR.
						13 * 30	230 mmHg	Inflated during the exercise and deflated during the rest periods	Volitional failure (4 * 22–36 repetitions)				
Laurentino et al. (2008)	23.55 ± 3.37	71.44 ± 11.05		8	80% 1RM	-	-	-	3–5 sets	2 min between set.	8	2	CSA; 1RM.
					80% 1RM	14 * 90	125.6 ± 15.0	Inflated during the exercise and deflated during the rest periods					
	22.42 ± 3.41	80.15 ± 11.758		8	60% 1RM	-	-	-	3–5 sets				
					60% 1RM	14 * 90	131.2 ± 12.8	Inflated during the exercise and deflated during the rest periods					
Laurentino et al. (2022)	23.6 ± 6	73.8 ± 12		10	80% 1RM	-	-	-	4 * 8	1.5 min between set.	8	2	CSA; 1RM; Hormones; Lactate concentration
	20.0 ± 4.5	72.1 ± 11.9		9	20% 1RM	17.5 * 92	80% of predicted arterial occlusion pressure (94.8 ± 10.3 mmHg)	Inflated for the entire exercise protocol	4 * 15	1 min between set.			
Lixandrão et al. (2015)	29.2 ± 9.9	74.9 ± 7.7	24.6 ± 2.7	9	80% 1RM	-	-	-	2–3 * 10	1 min between set.	12	2	CSA; 1RM.
	26.1 ± 7.6	80.6 ± 19.7	25.9 ± 5.6	11	20% 1RM	9.2 * 17.5	40% occlusion pressure (55.5 ± 7.6)	Inflated for the entire exercise protocol	2–3 * 15				
	28.9 ± 8.7	75.3 ± 10.7	24.6 ± 3.3	14	20% 1RM	9.2 * 17.5	80% occlusion pressure (109.6 ± 9.4)	Inflated for the entire exercise protocol	2–3 * 15				
	26.1 ± 7.6	74.7 ± 9.5	24.7 ± 2.1	8	40% 1RM	9.2 * 17.5	40% occlusion pressure (54.5 ± 4.6)	Inflated for the entire exercise protocol	2–3 * 15				
	28.9 ± 9.2	78.9 ± 20.7	25.0 ± 5.8	10	40% 1RM	9.2 * 17.5	80% occlusion pressure (105.0 ± 18.5)	Inflated for the entire exercise protocol	2–3 * 15				
Madarame et al. (2008)	21.9 ± 4.2	60.7 ± 5.1		7	30% 1RM	4 * 175	-	-	30/15/15/15	0.5 min between set.	10	2	CSA; 1RM.
	21.6 ± 2.4	58.8 ± 3.8		8	30% 1RM		160–240 mmHg	Inflated for the entire exercise protocol					
Ozaki et al. (2013)	24 ± 1	62.3 ± 2.9	21.4 ± 0.8	9	75% 1RM	-	-	-	3 * 10	2–3 min between set.	6	3	CSA; 1RM; carotid arterial compliance; resting blood pressure
	23 ± 0	63.9 ± 2.4	21.7 ± 0.8	10	30% 1RM	3	80–130 mmHg	Inflated for the entire exercise protocol	30/15/15/15	0.5 min between set.			
Reece et al. (1985)	22.34 ± 3.34	79.49 ± 16.19		15	80% of 1RM	-	-	-	Volitional failure	2 min between set.	6	3	CSA; 1RM; muscle fiber type; volume load
	21.35 ± 2.71	70.41 ± 12.52		15	30% of 1RM	10	50% AOP	Inflated for the entire exercise protocol		1 min between set.			
Teixeira et al. (2022)	26 ± 4	82.6 ± 9.4		5	30% of 1RM	-	-	-	3 * 15	1 min of rest between sets	3	2	CSA; 1RM.
					30% of 1RM	9 * 18	80% of AOP - 102 mmHg	Inflated for the entire exercise protocol					
Vechin et al. (2015)	62.0 ± 3.0	68.7 ± 15.3		8	70%–80% of 1RM	-	-	-	4 * 10	1 min of rest between sets	12	2	CSA; 1RM.
	65.0 ± 2.0	79.3 ± 17.9		8	20%–30% of 1RM	18	50% of AOP - 71 ± 9 mmHg	Inflated for the entire exercise protocol	30/15/15/15				
Yasuda et al. (2014)	67.7 ± 6.0	53.4 ± 9.1	21.3 ± 2.9	10	-	-	-	-	-	-	-	-	CSA; 1RM; arterial function tests (resting blood pressure et.); blood sampling and biochemical analyses
	71.3 ± 7.1	53.4 ± 9.3	20.8 ± 2.6	19	20%–30% of 1RM	5	270 mmHg	Inflated for the entire exercise protocol	30/20/15/10	0.5 min rest between each series (knee extension) 1.5 min rest between each series (leg press)	12	2	

“Training protocol” refers to the design of the resistance training program, including the number of sets and repetitions or the total number of repetitions for each exercise. “Rest” refers to the rest period between sets during each training session. “Duration (weeks)” indicates the length of the entire training program in weeks. “Frequency (per week)” denotes how often training sessions occur each week. “Outcomes” refer to the main results or findings of each study.

The current BFR training protocol is based on traditional resistance training protocol standards. The frequency of training sessions among the included studies varied between 2 and 4 times a week, with only one study implementing a training frequency of 4 times per week. Regarding the intensity of BFR combined with resistance training, a range of 20%–80% of 1 RM was observed among the intervention groups in the included studies. Among these groups, 23 intervention groups had intensities below 60% of 1 RM, indicating low-intensity resistance training (Schoenfeld et al., 2017). Only 2 intervention groups had intensities of 60%–80% of 1 RM, suggesting middle-intensity training. The remaining groups exhibited the BFR of 80% of 1RM, representing high-intensity training. Notably, the limited number of middle-intensity groups precluded specific meta-regression analysis. Therefore, these groups were amalgamated with the high-intensity groups as “other intensity with BFR” for further analysis. Considering the notable disparity in other trial characteristics across intervention groups, these were treated as covariates in subsequent meta-regression analyses.

It was observed that the intervention groups in the included studies utilized different types of tourniquets, with only one study (Brumitt et al., 2021b) omitting specific details regarding the tourniquet used. These tourniquets were tailored in length to match the cross-sectional area of participants’ arms or legs. Consequently, the present study analyzed the influence of tourniquet width on BFR training. In addition to blood pressure monitor’s accompanying pressurized belt, two different types of tourniquet products were applied in different studies, including the *Delphi Personalized Tourniquet System* with a tourniquet width of 11.43 cm in two studies (Bradley et al., 2022; Colapietro, 2023) and *Hokanson TD312 Calculating Cuff Inflator* with a tourniquet width of 6 cm in another two studies (Hackney et al., 2016; Cook and Cleary, 2019). In addition, 14 studies (20 intervention groups) had tourniquets inflated throughout training, and 6 studies (8 intervention groups) inflated the tourniquets during exercise and deflated them during the rest periods.

### 3.4 Effect estimates of BFR intervention to NOBFR intervention from meta-analysis

#### 3.4.1 Heterogeneity and possible publication bias

**FIGURE 3:**
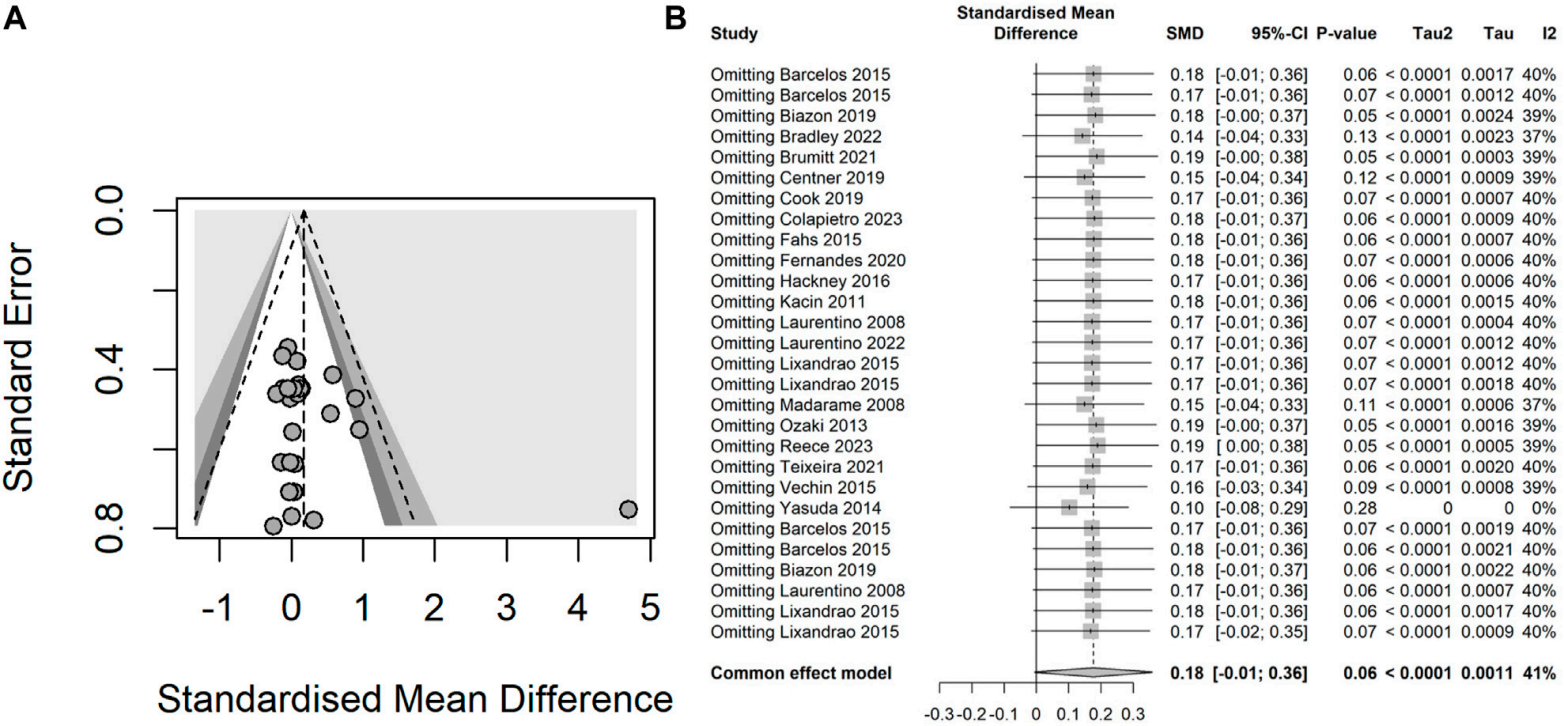
**(A)** Funnel plot illustrating the heterogeneity of studies within the intervention groups concerning muscle thickness results; **(B)** Forest plot demonstrating the effect on the overall robustness of the results upon the exclusion of each individual intervention for muscle thickness.

**FIGURE 4:**
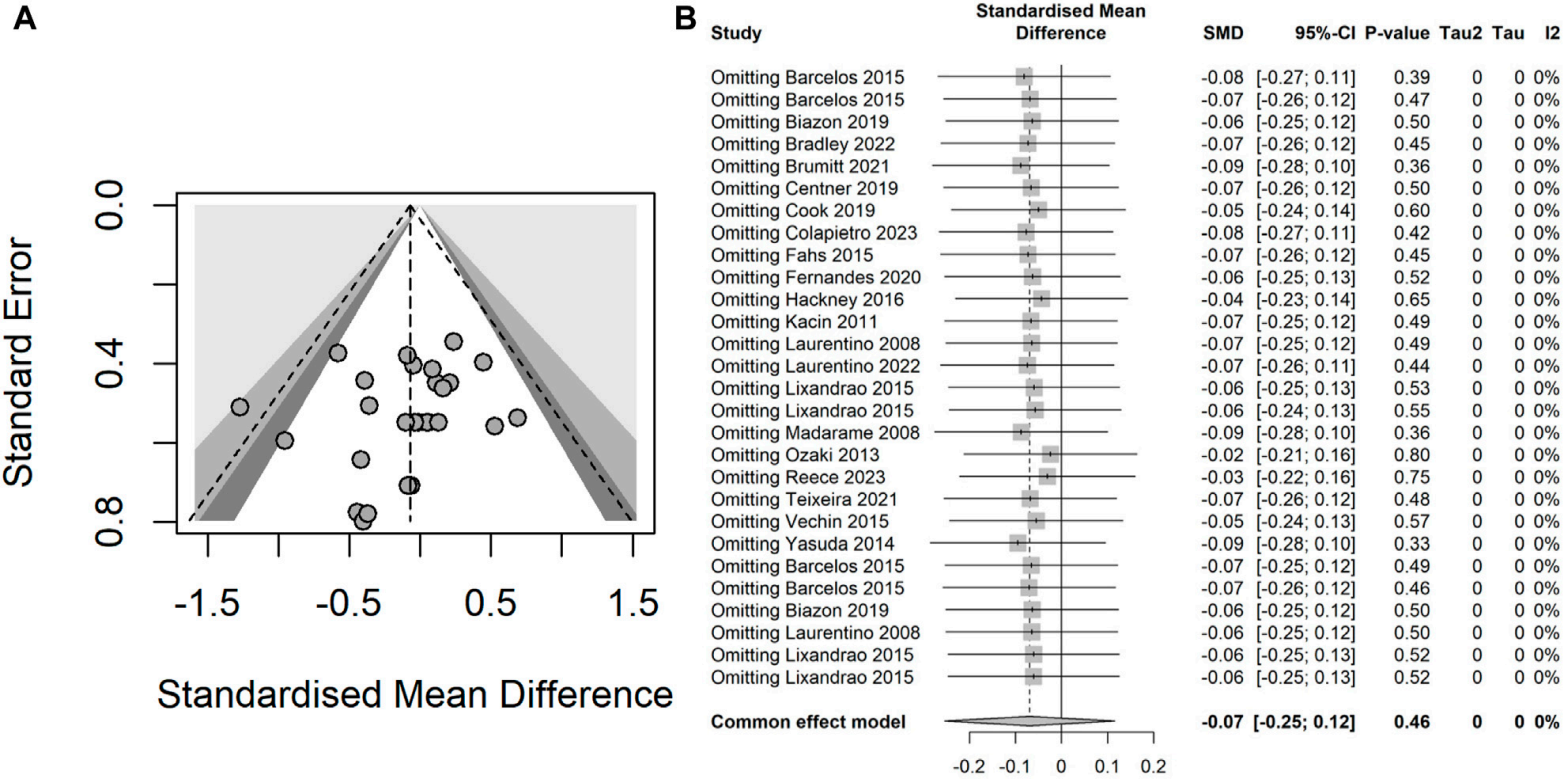
**(A)** Funnel plot illustrating the heterogeneity of studies within the intervention groups concerning muscle strength results; **(B)** Forest plot demonstrating the effect on the overall robustness of the results upon the exclusion of each individual intervention for muscle strength.

The fixed-effect and random-effects meta-analyses were performed to assess the overall heterogeneity. The assessment revealed a moderate heterogeneity (*I* ^*2*^ = 41%, *P* = 0.01) for muscle thickness results across the studies and a low heterogeneity (*I* ^*2*^ = 0%, *P* = 0.90) for the muscle strength results. The funnel plot of muscle thickness results highlighted significant heterogeneity in one studies (Yasuda et al., 2014) (Figure 3A), and the Egger’s test results indicated a lack of statistically significant asymmetry in the funnel plot (*t* = 1.43, *df* = 26, *P* = 0.16). Subsequently, the forest plot of sensitivity analysis showed that one study (Yasuda et al., 2014) significantly affected the robustness of the overall results, whereas two studies (Kacin and Strazar, 2011; Hackney et al., 2016) with a high risk assessed by RoB 2 did not impact the overall robustness (Figure 3B). Upon the exclusion of the study contributing to high heterogeneity, the overall heterogeneity reduced to a low level across all intervention groups (*I* ^*2*^ = 0%, *P* = 1.00) (Figure 4). Both the funnel plot and the forest plot of sensitivity analysis for muscle strength showed that none of the studies significantly affected the robustness of the overall results (Figures 4A, B). The Egger’s test did not detect significant asymmetry in the funnel plot.

#### 3.4.2 Subgroup meta-analysis of intervention intensity

**FIGURE 5:**
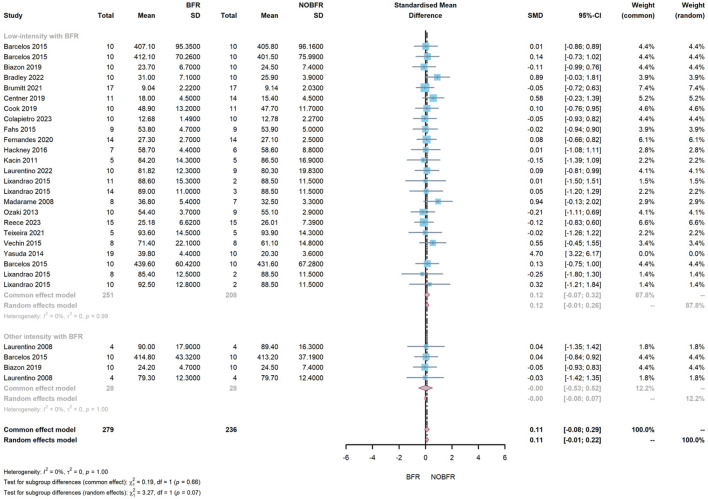
Forest plot demonstrating a subgroup meta-analysis assessing the effect of BFR combined with resistance training at low and other intensities on muscle thickness improvement.

The meta-analysis findings uncovered no significant discrepancy between resistance training with BFR and conventional resistance training in terms of enhancements in muscle thickness and strength. The overall contrast in muscle thickness improvement between BFR and conventional training stood at 0.11 (95% CI: −0.08–0.29, *I* ^*2*^ = 0%), indicating a no significance impact of conventional training on muscle thickness improvement. However, this difference was not statistically significant (*z* = 1.03, *P* = 0.302). Subgroup analysis revealed that BFR training combined with other intensity resistance training displayed a better effect on muscle thickness improvement (0.00, 95% CI: −0.53–0.52, *I* ^*2*^ = 0%) than BFR training with low-load resistance training (0.12, 95% CI: −0.07–0.32, *I* ^*2*^ = 0%). Nonetheless, this disparity failed to reach statistical significance ( ${ℵ}^{2}=0.19,df=1,p=0.66$ ), as shown in Figure 5.

**FIGURE 6:**
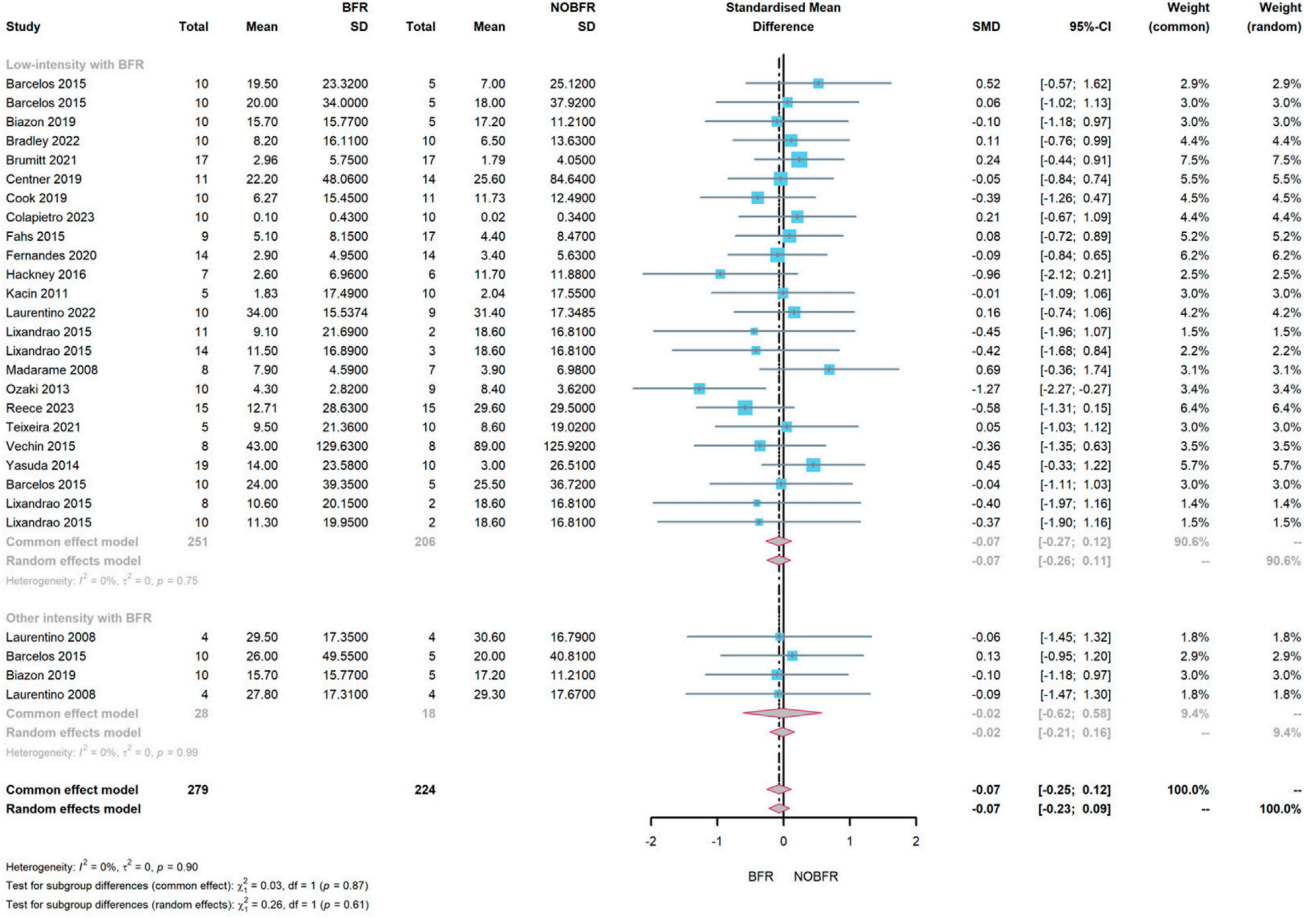
Forest plot illustrating a subgroup meta-analysis evaluating the effect of BFR combined with resistance training at low and other intensities on muscle strength improvement.

Regarding muscle strength improvement, the overall difference between BFR and routine resistance exercise was −0.07 (95% CI: −0.25–0.12, *I* ^*2*^ = 0%). This suggested that resistance exercise with BFR marginally surpassed routine resistance exercise in enhancing muscle strength (z = 0.74, P = 0.461). Subgroup meta-analysis showed that BFR combined with other intensity resistance exercise exhibited a better improvement effect on muscle strength (−0.02, 95% CI: −0.62–0.58, *I* ^*2*^ = 0%) than the low-intensity resistance exercise with BFR (−0.07, 95% CI: −0.27–0.12, *I* ^*2*^ = 0%). However, this difference did not reach statistical significance ( ${ℵ}^{2}=0.03,df=1,p=0.87$ ), as depicted in Figure 6.

#### 3.4.3 Meta-regression for BFR intervention

**FIGURE 7:**
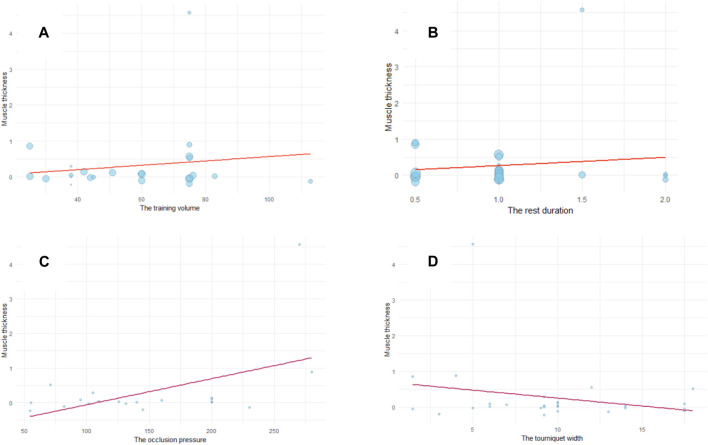
Meta-regression modelling of the effects of **(A)** training volume **(B)** training rest duration **(C)** tourniquet occlusion pressure, and **(D)** tourniquet width on muscle thickness improvement by BFR combined with resistance training.

The meta-regression analysis incorporated a total of 7 variables to examine their effects on muscle thickness and strength improvement (see Tables 4, 5). The regression models are presented in Figure 7. The meta-regression results indicated that none of the moderators exhibited statistically significant links with muscle thickness improvement (*P* > 0.05). Only the occlusion pressure of tourniquets showed a marginal effect on muscle thickness improvement (coefficient estimate = 0.006, 95% CI = 0–0.012, adjusted *R* ^*2*^ = 0.30), which was not statistically significant (*P* = 0.057). The regression model suggested a slight increase in muscle thickness with increased tourniquet occlusion pressure (see Figure 7). Conversely, the remaining moderators did not statistically significant improve muscle thickness. Furthermore, the moderators did not yield significant impacts on muscle strength improvement (*P* > 0.05. Adjusted *R* ^*2*^ = 0) (see Table 5).

**TABLE 4:** Meta-regression of moderators concerning the effects of BFR combined with resistance training on muscle thickness improvement.

Variable	Number of intervention groups	Coefficient	Std.err	*t*	*p*	95% CI	Adjusted *R* ^*2*^
Intervention intensity	28	−0.213	0.387	−0.55	0.587	−1.008, 0.582	0
Training volume	24	0.003	0.008	0.41	0.685	−0.013, 0.020	−42.62%
Rest duration	27	0.149	0.356	0.42	0.680	−0.584, 0.882	−94.62%
Training sessions	28	0.009	0.013	0.65	0.524	−0.019, 0.036	0
Tourniquet width	27	−0.026	0.028	−0.93	0.361	−0.084, 0.032	−79.63%
Occlusion pressure	20	0.006	0.003	2.03	0.057	0, 0.012	30.23%
Tourniquet application time	28	−0.164	0.287	−0.57	0.572	−0.753, 0.425	0

**TABLE 5:** Meta-regression of moderators concerning the effects of BFR combined with resistance training on muscle strength improvement.

Variable	Number of intervention groups	Coefficient	Std.err	*t*	*p*	95% CI	Adjusted *R* ^*2*^
Intervention intensity	28	0.054	0.322	0.17	0.869	−0.608, 0.716	0
Training volume	24	−0.001	0.005	−0.23	0.818	−0.011, 0.009	0
Rest duration	27	−0.046	0.238	−0.19	0.847	−0.537, 0.444	0
Training sessions	28	−0.002	0.010	−0.21	0.834	−0.023, 0.018	0
Tourniquet width	27	−0.004	0.020	−0.19	0.848	−0.045, 0.038	0
Occlusion pressure	20	0.004	0.002	2.09	0.051	0, 0.008	0
Tourniquet application time	28	−0.009	0.213	−0.04	0.966	−0.446, 0.428	0

## 4 Discussion

This meta-analysis aimed to assess the efficacy of muscle thickness and strength improvement by comparing resistance training combined with BFR to traditional resistance training. The comprehensive analysis revealed no significant difference between these two intervention methods. Notably, resistance training with BFR at other intensities (middle- and high-intensity) marginally outperformed low-intensity resistance with BFR in muscle strength and thickness improvement. However, no significant difference was noted in this regard between higher-intensity and lower-intensity resistance with BFR. These findings suggest that from a safety standpoint, higher-intensity resistance exercises with BFR might not be imperative for fragile populations. Moreover, the meta-regression results suggested a potential impact of tourniquet occlusion pressure on intervention outcomes, while trial characteristics extracted from literature review showed no significant association with muscle thickness and strength improvement. Nevertheless, these trial characteristics provide crucial reference information for establishing standards for BFR combined with resistance training in the future.

The meta-analysis results further revealed that resistance training coupled with BFR exerted a comparable influence to traditional resistance on muscle strength and thickness improvement. Although high-intensity resistance training stands out as the most efficacious method for enhancing muscle strength and thickness, its applicability is constrained by potential risks, especially for fragile populations. Several studies (Krzysztofik et al., 2019; Koc et al., 2022; Krzysztofik et al., 2022) have indicated that resistance training with BFR offers a promising alternative to traditional methods, demonstrating comparable effects. Older adults and hospitalized patients commonly grapple with muscle loss and weakness owing to a lack of exercise (Ruiz et al., 2008), often associated with a decline in type II muscle fibers (Park et al., 2022). High-intensity resistance training, although effective in improving muscle strength through neural adaptions, may pose injury risks, particularly for old adults or patients, given the acute hemodynamic response it triggers (Nascimento et al., 2022). The repeated elevation in blood pressure increases endothelial shear, thrombin, and fibrin (Hansen et al., 2022), heightening the risk of venous thrombosis (Hansen et al., 2022). In contrast, resistance training coupled with BFR stimulates muscular hypertrophy by recruiting type II muscular fibers due to the localized hypoxia environment, thus enhancing muscle strength (Pour et al., 2017). This method, nevertheless, has also been reported to elevate blood pressure and provoke certain atypical vascular reactions (Nascimento et al., 2022), such as ischemia-reperfusion injury (Cristina-Oliveira et al., 2020). However, a recent study (Nascimento et al., 2019) reported contrasting findings, suggesting that resistance training with BFR did not induce coagulation activity; instead, it demonstrated an increase in fibrinolytic activity. This suggests that this intervention may not pose an elevated risk of thrombus formation. In addition, resistance training with BFR may have potential effects on endothelial function and vascular regeneration (Zhang et al., 2022), although further evidence is needed for confirmation. Despite these considerations, resistance training with BFR has already been applied in some clinical settings for functional rehabilitation (Hughes et al., 2019; Killinger et al., 2020; Anandavadivelan et al., 2024). However, aspects such as training intensity and some trial characteristics necessitate further elucidation.

The standardization of resistance training protocols with BFR remains elusive, with factors like training intensity, duration, and tourniquets specifications during the intervention potentially influencing intervention effectiveness. In view of this, this study conducted subgroup meta-analysis and meta-regression to examine the relationships between trial characteristics and muscle hypertrophy. The subgroup analysis aimed at assessing the influence of different intensities on muscle changes. Notably, limited research has explored the effects of other intensities, especially high-intensity resistance training with BFR, on muscle changes, underscoring the necessity for further validation of its impact. Among the studies included in our analysis, only three (comprising four intervention groups) implemented moderate- (60%–80% of 1 RM) and high-intensity (over 80% of 1 RM) resistance training with BFR. Subgroup analysis was conducted to compare the effects of low-intensity (below 60% of 1 RM) resistance training with BFR against other intensities (moderate and high intensities) of resistance training with BFR, aiming to elucidate the differential effects on muscle changes. The results revealed no significant difference in muscle strength and thickness improvements between low-intensity and moderate- and high-intensity resistance training with BFR. Studies by Biazon et al. (2019); Laurentino et al. (2008) reported that high-intensity resistance training with BFR did not notably outperform high-intensity resistance training without BFR in inducing muscle hypertrophy. However, Barrett, 2017 discovered that 70% of 1 RM resistance training with BFR did not lead to greater muscle hypertrophy than moderate-to-intensity training alone during a 12-week training period. This discrepancy might be attributed to participant characteristics, as Barrett’s study involved highly trained males, potentially limiting substantial gains in muscle strength and thickness compared to untrained populations. Differing trial characteristics, such as trial volume, might also play a role in this divergence. Barrent’s study Barrett (2017) adjusted the training volume based on participants’ individual conditions, such as premature fatigue; in contrast, studies by Biazon et al. (2019); Laurentino et al. (2008) employed a fixed training protocol for each participant.

Further substantiation is required to affirm the impact of moderate- and high-intensity resistance training with BFR on muscle hypertrophy. Compared to low-intensity resistance training with BFR, high-intensity resistance training with BFR presnets heightened challenges to participants’ physical condition and exerts greater stress on the cardiovascular system, potentially posing risk during training (Hansen et al., 2022). In light of current findings, advocating for the application of low-intensity resistance training with BFR in clinical settings and for populations unable to endure high-intensity training seems prudent. Bradley et al. (2022) demonstrated that low-load BFR training (30% 1 RM) resulted in lower Rating of Perceived Exertion (RPF) scores than training at 70% 1RM. For individuals lacking training experience or unable to withstand high-intensity training, implementing low-load training with BFR may alleviate discomfort during training. Additionally, low-intensity resistance training with BFR exerts notably less strain on joints and soft tissues than high-intensity resistance training (Patterson et al., 2019). It is worth noting that 20% 1 RM is equivalent to the intensity of daily activities (Almeida et al., 2022), but studies involving training intensities below 20% 1 RM are currently lacking. Training at intensities lower than 20% 1 RM may not induce sufficient biological pressure to stimulate muscle hypertrophy. Therefore, low-intensity resistance training with BFR is recommended for daily training due to its safety and effectiveness.

The meta-regression results revealed an absence of significant impact from the training protocol characteristics on the outcomes. This observation may be attributed to the minor variations in training protocols among the studies included in our analysis. The intervention protocols in these studies were derived from modifications to traditional resistance training foundation, prescribing a training frequency of 2-3 sessions per week with a minimum duration of 4 weeks. Notably, evidence from several studies suggests that this approach can elicit muscle hypertrophy within 2 weeks (Hill et al., 2018; Teixeira et al., 2022). Six studies adhered consistently to a specific design for BFR training sets and repetitions of 30/15/15/15 (four sets). In addition to the details of the training protocols, certain characteristics of the tourniquet used during training may also wield influence over the intervention outcomes.

In addition to training intensity, our meta-regression explored the link between certain tourniquets characteristics and their effects on muscle hypertrophy. The analysis unvialed no significant association between deflating the tourniquet during rest periods and its impact on muscle hypertrophy. However, these findings were drawn from trials focusing on LI resistance training with BFR, with limited representation of other-intensity resistance training with BFR. Consequently, the existing evidence might not be sufficient to establish a definitive relationship between tourniquet inflation status and the effects of high-intensity resistance training with BFR on muscle hypertrophy. Laurentino et al. (2008), for instance, reported discomfort and early fatigue associated with inflated tourniquets during high-intensity resistance training, potentially influencing muscle hypertrophy improvements (Das and Paton, 2022). The practice of deflating tourniquets during rest periods may contribute to maintaining adequate training volume for muscle hypertrophy in certain contexts (Barrett, 2017).

The width and occlusion pressure of tourniquets have surfaced as potential influencers of intervention efficacy. Presently, there are no standardized criteria for tourniquet usage in BFR training. Among the literature included, only two companies offered blocking tourniquet products tailored for training, each with differing specifications. While tourniquet width did not exhibit a significant association with muscle changes, it potentially impacted the intervention’s effectiveness. The regression model indicated slight reduction in intervention improvement with an increase in tourniquets width. Although direct investigation into the effect of tourniquet width on intervention effectiveness was lacking, previous research suggested that wider tourniquets may exert greater occlusion pressure due to decreased pressure required to occlude blood vessels. Loenneke et al. (2012) observed that the pressure required to occlude blood flow decreases proportionally with increasing cuff width. However, it is essential to note that these results may be influenced by the cuff material in the study, as narrower tourniquets demonstrated better elasticity compared to wider ones. Thus, wider tourniquets might induce better localized hypoxia, potentially enhancing muscle growth stimulation (Michal et al., 2020). The studies encompassed in our analysis did not account for different occlusion pressures resulting from varying widths, which could potentially affect the intervention’s effectiveness. This emphasizes the need for future research to explore the synergistic effects of tourniquet width and pressure, as well as the influence of tourniquet material on intervention outcomes. Additionally, considering that a narrower cuff may lead to localized muscle damage due to increased strain (Das and Paton, 2022), it follows that a wider cuff would be more practical for application.

Regarding the pressure applied by tourniquets, the results of the meta-regression indicated that it did not exhibit a significant association with intervention outcomes. However, the regression model highlighted that increased occlusion pressure correlated with more effective muscle hypertrophy improvement. Higher restriction pressure (70%–100% LOP) may indicate a lower blood supply, leading to increased metabolic strain during exercise, potentially resulting in an enhanced muscle strength and hypertrophy (Fabero-Garrido et al., 2022b; Cognetti et al., 2022). Nevertheless, careful selection of the occlusion pressure is crucial. Most studies in our analysis utilized an arterial occlusion pressure (AOP) ranging from 40% to 80%. It has been suggested that an AOP of 50%–80% is optimal for BFR training (Das and Paton, 2022), and an AOP exceeding 80% may elevate the risk of adverse events during patient training, such as the potential induction of venous thrombosis (Stavres et al., 2018). Furthermore, higher cuff pressure may lead to discomfort, post-exercise soreness, and reduced total exercise volume. Notably, 8 studies (40%) included in our analysis did not apply individualized pressure settings to participants, potentially resulting in training-related pain (Koc et al., 2022). Hence, careful consideration regarding the width and occlusion pressure of tourniquets is vital during training interventions to minimize potential adverse effects while achieving desired muscle growth.

## 5 Conclusion

Low intensity resistance training with BFR is a safe and effective training program. This training method induces muscle hypertrophy in a short period of time and alleviates the process of muscle wasting by stimulating and engaging type II muscle fibre contractions. It also reduces the risk of venous thrombosis. However, it is important to note that although this training programme is similar to high-intensity resistance training, it may place a greater cardiovascular workload, which may lead to adverse events. In order to improve the improvement in muscle strength and dimensions with this intervention protocol, this study analysed the results of existing studies. Based on the results, this study suggests that the prescription of the programme could be guided by the following parameters: 2-3 training sessions per week at an intensity of 20%–40% of 1 RM. In addition, it is recommended to use a wider cuff and to apply 50%–80% of the arterial occlusion pressure during training to stimulate improvements in muscle strength and thickness. In addition, releasing tourniquet pressure during rest periods is beneficial to reduce participant discomfort during training.

While our investigation provides valuable insights, the limited number of studies examining the combination of high-intensity resistance training and BFR restricts the generalization of our findings, particularly in the context of high-intensity training. Furthermore, our findings highlight the need for further exploration into the effects of tourniquet width and pressure on intervention outcomes. Subsequently, studies should endeavor to investigate the synergistic effects of tourniquet pressure and width on intervention effects. Additionally, the characteristics of tourniquet materials, such as elasticity, and their impact on intervention outcomes remain an understudied area that warrants attention.

## Data Availability

The original contributions presented in the study are included in the article/Supplementary Material, further inquiries can be directed to the corresponding author.
