# Supplementary material for: Blood flow restriction combined with resistance training on muscle strength and thickness improvement in young adults: a systematic review, meta-analysis, and meta-regression
Source: Front Physiol. 2024 Aug 12;15:1379605. doi: 10.3389/fphys.2024.1379605 (PMC11345148; doi:10.3389/fphys.2024.1379605)
Supplement: Supplementary file 1 [file Table1.docx]

**Appendix**

Table 1 Example of study search strategy and results using PubMed Database.

| Search number | Query | Sort By | Filters | Search Details | Results | Time |
| --- | --- | --- | --- | --- | --- | --- |
| 5 | (#3) AND (#4) |  |  | ("Resistance Training"[MeSH Terms] OR "strength training"[Title/Abstract] OR "resistance exercise"[Title/Abstract] OR "weight training"[Title/Abstract]) AND ("Hypoxia"[MeSH Terms] OR "altitude"[Title/Abstract] OR "hypoxic training"[Title/Abstract] OR "hypoxic exposure"[Title/Abstract] OR ("Blood Flow Restriction Therapy"[MeSH Terms] OR "kaatsu"[Title/Abstract] OR "occlusion training"[Title/Abstract] OR "blood flow restriction"[Title/Abstract] OR "bfr training"[Title/Abstract] OR "bfr exercise"[Title/Abstract])) | 695 | 22:37:31 |
| 4 | (#1) OR (#2) |  |  | "Hypoxia"[MeSH Terms] OR "altitude"[Title/Abstract] OR "hypoxic training"[Title/Abstract] OR "hypoxic exposure"[Title/Abstract] OR "Blood Flow Restriction Therapy"[MeSH Terms] OR "kaatsu"[Title/Abstract] OR "occlusion training"[Title/Abstract] OR "blood flow restriction"[Title/Abstract] OR "bfr training"[Title/Abstract] OR "bfr exercise"[Title/Abstract] | 116,016 | 22:37:08 |
| 3 | "Resistance Training"[MeSH Terms] OR "strength training"[Title/Abstract] OR "resistance exercise"[Title/Abstract] OR "weight training"[Title/Abstract] |  |  | "Resistance Training"[MeSH Terms] OR "strength training"[Title/Abstract] OR "resistance exercise"[Title/Abstract] OR "weight training"[Title/Abstract] | 21,681 | 22:36:23 |
| 2 | "Blood Flow Restriction Therapy"[MeSH Terms] OR "kaatsu"[Title/Abstract] OR "occlusion training"[Title/Abstract] OR "blood flow restriction"[Title/Abstract] OR "bfr training"[Title/Abstract] OR "bfr exercise"[Title/Abstract] |  |  | "Blood Flow Restriction Therapy"[MeSH Terms] OR "kaatsu"[Title/Abstract] OR "occlusion training"[Title/Abstract] OR "blood flow restriction"[Title/Abstract] OR "bfr training"[Title/Abstract] OR "bfr exercise"[Title/Abstract] | 1,187 | 22:35:48 |
| 1 | "Hypoxia"[MeSH Terms] OR "altitude"[Title/Abstract] OR "hypoxic training"[Title/Abstract] OR "hypoxic exposure"[Title/Abstract] |  |  | "Hypoxia"[MeSH Terms] OR "altitude"[Title/Abstract] OR "hypoxic training"[Title/Abstract] OR "hypoxic exposure"[Title/Abstract] | 114,864 | 22:33:27 |

Table 2 Study Quality assessment using RoB2

| Study | Randomization process | Deviations from intended interventions | Mising outcome data | Measurement of the outcome | Selection of the reported result | Overall Bias |
| --- | --- | --- | --- | --- | --- | --- |
| Barcelos 2015 | Low | Low | Low | Low | Low | Some concerns |
| Biazon 2019 | Low | Low | Low | Low | Low | Low |
| Bradley 2022 | Low | Low | Low | Low | Low | Low |
| Brumitt 2021 | Low | Low | Some concerns | Low | Low | Some concerns |
| Centner 2019 | Low | Low | Low | Low | Low | Low |
| Cook 2019 | Low | Low | Low | Low | Low | Low |
| Colapietro 2023 | Low | Low | Low | Low | Low | Low |
| Fahs 2015 | Low | Some concerns | Low | Low | Low | Some concerns |
| Fermandes 2020 | Low | Low | Low | Low | Low | Low |
| Hackney 2016 | High | Low | Low | Low | Low | High |
| Kacin 2011 | High | Low | Low | Low | Low | High |
| Laurentino 2008 | Some concerns | Low | Low | Low | Low | Some concerns |
| Laurentino 2022 | Low | Low | Some concerns | Low | Low | Some concerns |
| Lixandrao 2015 | Some concerns | Low | Some concerns | Low | Low | Some concerns |
| Madarame 2008 | Low | Low | Low | Low | Low | Low |
| Ozaki 2012 | Low | Low | Low | Low | Low | Low |
| Reece 2023 | Low | Low | Low | Low | Low | Low |
| Teixeira 2021 | Low | Low | Some concerns | Low | Low | Some concerns |
| Vechin 2015 | Some concerns | Low | Some concerns | Low | Low | Some concerns |
| Yasuda 2014 | Low | Low | Low | Low | Low | Low |

Table 3 Study quality assessment using the PEDro scale

| Study | 1 | 2 | 3 | 4 | 5 | 6 | 7 | 8 | 9 | 10 | 11 | Overall | Quality |
| --- | --- | --- | --- | --- | --- | --- | --- | --- | --- | --- | --- | --- | --- |
| Barcelos 2015 | 1 | 1 | 1 | 1 | 0 | 0 | 0 | 1 | 1 | 1 | 1 | 8/11 | High |
| Biazon 2019 | 1 | 1 | 1 | 1 | 0 | 0 | 0 | 1 | 1 | 1 | 1 | 8/11 | High |
| Bradley 2022 | 1 | 1 | 1 | 1 | 0 | 0 | 1 | 0 | 1 | 1 | 1 | 8/11 | High |
| Brumitt 2021 | 1 | 1 | 1 | 1 | 0 | 0 | 1 | 1 | 1 | 1 | 1 | 9/11 | High |
| Centner 2019 | 1 | 1 | 1 | 1 | 0 | 0 | 1 | 1 | 1 | 1 | 1 | 9/11 | High |
| Cook 2019 | 0 | 1 | 1 | 1 | 0 | 0 | 0 | 1 | 1 | 1 | 1 | 7/11 | Some concern |
| Colapietro 2023 | 1 | 1 | 1 | 1 | 0 | 0 | 0 | 1 | 1 | 1 | 1 | 8/11 | High |
| Fahs 2015 | 1 | 1 | 1 | 1 | 0 | 0 | 0 | 0 | 1 | 1 | 1 | 7/11 | Some concern |
| Fernandes 2020 | 1 | 1 | 1 | 1 | 0 | 0 | 0 | 1 | 1 | 1 | 1 | 8/11 | High |
| Hackney 2016 | 1 | 0 | 1 | 1 | 0 | 0 | 0 | 1 | 1 | 1 | 1 | 7/11 | Some concern |
| Kacin 2011 | 1 | 0 | 1 | 1 | 0 | 0 | 0 | 1 | 1 | 1 | 1 | 7/11 | Some concern |
| Laurentino 2008 | 1 | 0 | 1 | 1 | 0 | 0 | 0 | 1 | 1 | 1 | 1 | 7/11 | Some concern |
| Laurentino 2022 | 1 | 1 | 1 | 1 | 0 | 0 | 0 | 1 | 1 | 1 | 1 | 8/11 | High |
| Lixandrão 2015 | 1 | 0 | 1 | 1 | 0 | 0 | 0 | 0 | 1 | 1 | 1 | 6/11 | Some concern |
| Madarame 2008 | 0 | 1 | 1 | 1 | 0 | 0 | 0 | 1 | 1 | 1 | 1 | 7/11 | Some concern |
| Ozaki 2013 | 1 | 1 | 1 | 1 | 0 | 0 | 0 | 1 | 1 | 1 | 1 | 8/11 | High |
| Reece 2023 | 1 | 1 | 1 | 1 | 0 | 0 | 0 | 1 | 1 | 1 | 1 | 8/11 | High |
| Teixeira 2021 | 1 | 1 | 1 | 1 | 0 | 0 | 0 | 0 | 1 | 1 | 1 | 7/11 | Some concern |
| Vechin 2015 | 1 | 0 | 1 | 1 | 0 | 0 | 0 | 1 | 1 | 1 | 1 | 7/11 | Some concern |
| Yasuda 2014 | 1 | 1 | 1 | 1 | 0 | 0 | 0 | 1 | 1 | 1 | 1 | 8/11 | High |
